# Molecular screening in a translational large animal trial identifies a differential inflammatory response for MINOCA

**DOI:** 10.1007/s00395-025-01118-9

**Published:** 2025-06-13

**Authors:** Jasper Iske, Joshua M. Mesfin, Petra Wolint, Miriam Weisskopf, Christien Beez, Henriette Thau, Christian T. Stoeck, January M. Weiner, Melanie M. Hierweger, Eva van Gelder, Thorald Stolte, Nuri Ünesen, Ross Straughan, Lucas S. J. Eckholt, Nina Trimmel, Dieter Beule, Heike Meyborg, Timo Z. Nazari-Shafti, Volkmar Falk, Maximilian Y. Emmert, Nikola Cesarovic

**Affiliations:** 1 Department of Cardiothoracic and Vascular Surgery, Deutsches Herzzentrum Der Charité (DHZC), Berlin, Germany; 2 Charité – Universitätsmedizin Berlin, Corporate Member of Freie Universität Berlin and Humboldt-Universität zu Berlin and Berlin Institute of Health, Berlin, Germany; 3 Berlin Institute of Health at Charité-Universitätsmedizin Berlin, Berlin, Germany; 4 DZHK (German Centre for Cardiovascular Research), Partner Site, Berlin, Germany; 5 Department of Health Sciences and Technology, ETH Zürich, Leopold-Ruzicka-Weg 4, HCP H 12.1, Zurich, Switzerland; 6 Center for Preclinical Development, University Hospital of Zürich, University of Zürich, Zurich, Switzerland; 7 BIH Center for Regenerative Therapies (BCRT), Berlin Institute of Health at Charité-Universitätsmedizin Berlin, 13353 Berlin, Germany; 8 Michael E. DeBakey Department of Surgery, Baylor College of Medicine, Houston, TX USA; 9 Institute for Regenerative Medicine (IREM), University of Zürich, Zurich, Switzerland

**Keywords:** Microcirculation, MINOCA, MI, Inflammation, Thrombosis, Leukotriene

## Abstract

Myocardial infarction without obstructive coronary arteries (MINOCA) comprises up to 15% of all myocardial infarctions (MI) and could be caused by cardiac microembolization (CME) originating from plaque rupture and/or erosion. Early diagnosis remains a challenge due to limited early biomarkers, leading to high morbidity. Here, we have systematically characterized acute (up to 5 h) CME-induced MINOCA in comparison to MI using clinical markers, histology, multi-ELISAs, miRNA profiling, and proteomics in a translational porcine animal model. CME-induced MINOCA model was created by injecting autologous microthrombi, generated by carotid crush maneuver, into the coronary arteries, whereas MI was induced by LAD balloon occlusion/reperfusion. MINOCA animals exhibited low troponin (547.0 ± 489.2 ng/L) and creatine kinase (1827.8 ± 677.3 U/L) levels, as well as infarct size (2.3 ± 0.8%), necrosis (7.6 ± 3.2%), and interstitial hemorrhage (0.6 ± 0.4%). Immune cell infiltration surrounding MINOCA microthrombi sites was significantly higher (1532 ± 722 cells/mm^2^) in comparison to MI infarct zones (470 ± 320 cells/mm^2^). Furthermore, cytokine profiling showed elevated IL-1α and IL-1β in both groups, higher IL-10 in MINOCA, and higher IFN-y in MI. The MINOCA-specific pro-inflammatory miRNA, ssc-miR-802, was identified. Plasma proteomic analysis revealed leukotriene signaling as a MINOCA inflammatory pathway with augmented leukotriene-A4-hydrolase levels. Its product, leukotriene B4, was increased in MINOCA serum at 150 min (1031 ± 537.6 pg/mL) and 300 min (1309 ± 640.8 pg/mL) and in tissue (408.2 ± 92.12 pg/mL) vs. MI (428.9 ± 9.483 pg/mL in serum at 150 min, 308.76 ± 5.484 pg/mL in serum at 300 min, and 76.22 ± 31.12 pg/mL in tissue). In summary, CME-induced MINOCA elicits a distinct pro-inflammatory leukotriene response compared to MI, presenting a new acute MINOCA diagnostic and therapeutic target.

**Supplementary Information:**

The online version contains supplementary material available at 10.1007/s00395-025-01118-9.

## Introduction

Cardiac microembolization (CME) can result from coronary plaque rupture and/or erosion and present as acute coronary syndrome (ACS). Typically, no flow-limiting coronary obstruction (> 50%) is seen on angiography in major epicardial vessels, leading to the classification of these events as MINOCA, myocardial infarction with non-obstructive coronary arteries [5, 45]. MINOCA accounts for up to 15% of all MI cases, with plaque erosion and subsequent CME constituting the major pathology [44, 58]. MINOCA patients differ from those with MI, as MINOCA is associated with unspecific ECG alterations and lower troponin levels [35, 45]. According to the 2023 guidelines of the European Society for Cardiology, MINOCA is defined and diagnosed based on symptoms, electrocardiogram (ECG), coronary computed tomography angiography and cardiac magnetic resonance imaging (CMR) [5]. CMR represents the most sensitive method for diagnosis but is difficult to integrate in the diagnostic workup in the setting of ACS. This often leads to a delay of diagnosis of MINOCA in patients presenting with ACS, which marks the foundation for any therapeutic intervention. As a consequence, MINOCA patients are less likely to receive cardioprotective medications when compared to patients with MI [1]. In addition, no targeted therapy approaches have yet been established for MINOCA due to insufficient characterization of the pathophysiological processes and multiple causes underlying this disease. As a result, MINOCA is associated with a high risk for major adverse cardiac events leading to early re-hospitalization, with an in-hospital and 5-year mortality rate of 4.57% and 10.9%, respectively [30].

With these factors in mind, clinical trials have been conducted to uncover clinical markers of MINOCA. Recently, a post hoc study of the PLATO trial has evaluated MINOCA and MI patients with clinical biomarkers up to 1 month after index event [25]. While MINOCA has had lower troponin levels compared to MI, the MINOCA patients displayed elevated clinical markers of inflammation at early timepoints, suggesting that the acute inflammatory responses may be useful to leverage for MINOCA therapies [25]. However, the exact pathways leading to higher inflammation levels in MINOCA are unknown. Other studies have confirmed that higher level of inflammatory processes, as measured by higher cytokine levels [53], have been observed up to 3 months after diagnosing MINOCA [26]. While these trials provide a great foundation for uncovering diagnostic biomarkers and therapeutic targets for MINOCA, there are significant limitations in terms of generating a mechanistic understanding of this disease. Collecting multiple serum and tissue samples during myocardial infarction is not attainable in a clinical trial due to patient safety and ethical reasons. On the other hand, the implementation of clinical grade devices, equipment and diagnostic approaches allow for precise mimicking of the clinical setting in translational large animal models. Hence, there is an urgent and unmet medical need to characterize MINOCA pathophysiology, to identify key disease driving processes, to develop specific biomarkers for early diagnosis and to identify novel therapeutic targets in an animal model-based experimental platform.

Since the investigation of molecular processes underlying MINOCA and MI in clinical trials remains challenging due to the out-of-hospital nature of index events, comorbidities and ethical constraints, coronary microembolization models are a good option for recapitulating the inflammatory response and patchy infarcts present in MINOCA [32, 52]. Previous studies on this topic suggest that the main mechanism responsible for cardiomyocyte death and contractile dysfunction in MINOCA is a strong inflammatory response triggered by microembolization, rather than ischemia as a result of macrovascular obstruction [32]. Moreover, contractile dysfunction was ameliorated with glucocorticoids, an anti-inflammatory treatment [52]. Specifically, alongside nitric oxide and sphingosine [57], TNF-α signaling was hereby shown as a main driver of myocardial dysfunction [10, 31, 51]. However, TNF-α has played a dual role in coronary microembolization, also leading to delayed protection against infarction [51]. Administration of TNF-α neutralizing antibodies demonstrates this dual phenomenon, with improved contractile dysfunction and systolic wall thickening but no infarct size reduction [11, 24, 51]. In addition, TNF-α has played a role in potentiating serotonin’s vasoconstrictive properties [31], potentially leading to reduced coronary flow reserve as found in other large animal microembolization models and in MINOCA patients [23, 50]. Thus, inflammatory markers play a central role in MINOCA pathology. Circulating miRNAs have also been used as biomarkers for CME-induced injury [9, 54], eliciting downstream inflammatory markers found in inducing myocyte damage.

**Fig. 1:**
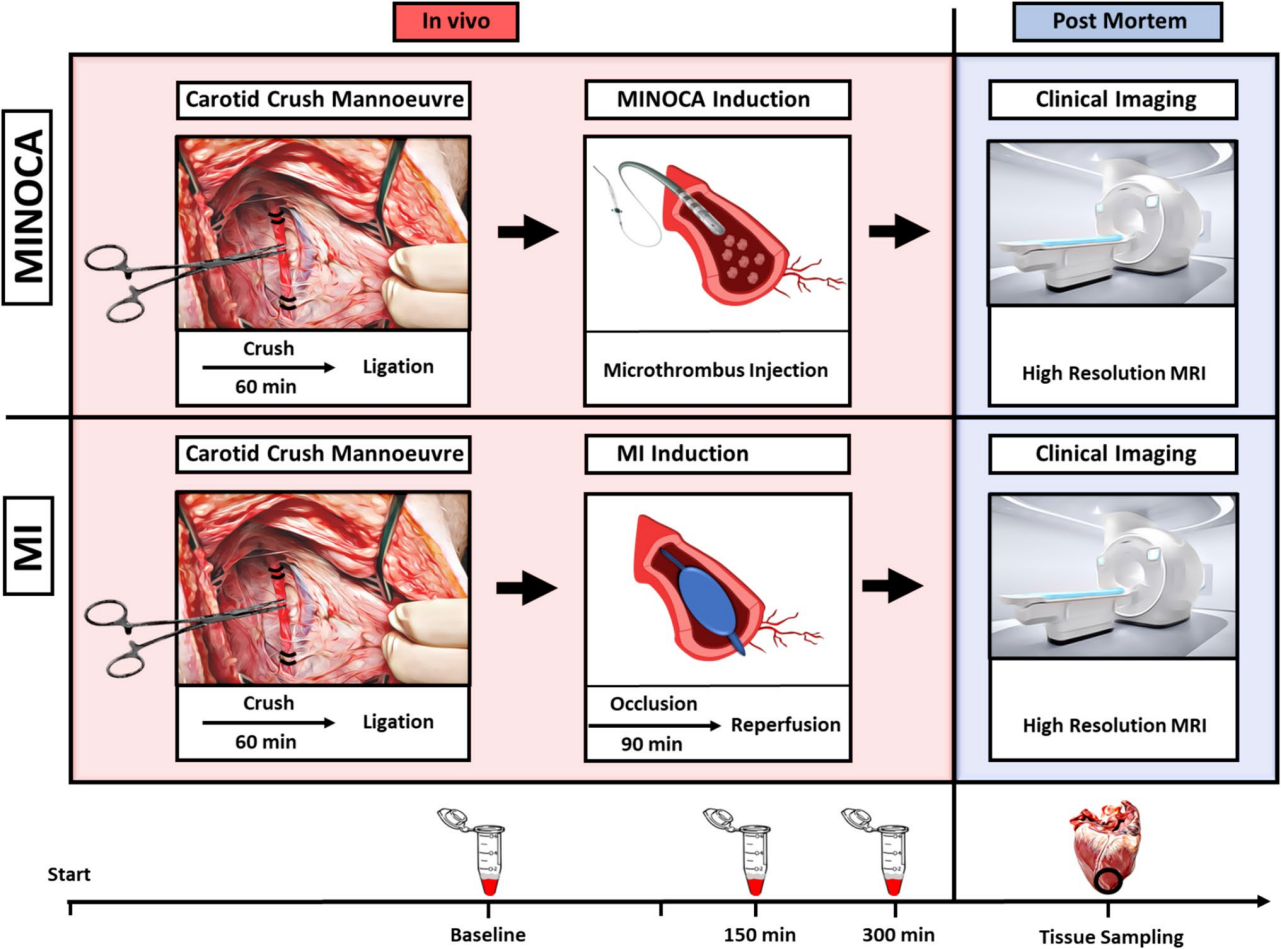
Schematic illustration of the animal model and sampling strategy. To mimic MINOCA, a carotid crush maneuver was performed to generate microthrombi which were subsequently injected into the coronary artery to induce CME. To reduce confounders, MI animals also received carotid crush, but no microthrombi were injected. MI animals were subjected to 90 min of coronary artery balloon occlusion followed by reperfusion. After MINOCA/MI was induced, plasma samples were collected at 150 and 300 min post-procedure. Following euthanasia, a high-resolution MRI was performed in addition to extensive tissue sampling for histology and downstream molecular assays

However, mimicking CME by inert microsphere injection has been suggested to underestimate the true inflammatory response induced through microthrombi in the clinic [10]. Therefore, we have recently established a translational porcine animal model involving the injection of autologous microthrombi into the coronary circulation that resembles all clinical features of MINOCA [8]. Compared to inert microspheres, administering autologous microthrombi allows for better modeling of acute inflammatory progression and overall microvascular occlusion due to the biological interplay of autologous microthrombi with the vascular endothelium and inflammatory cells [12, 32, 47]. Thus, with MI as a comparison, we used this model to investigate systemic molecular and inflammatory patterns to uncover potential therapeutic and diagnostic markers for MINOCA (Fig. 1).

## Materials and methods

### Animal acquisition, health check, and anesthesia

The animal study was approved by the local Committee for Animal Experimental Research (Cantonal Veterinary Office Zürich, Switzerland) under the license number ZH213/2019 and included 15 domestic pigs (Sus scrofa domestica, breed: Swiss large white). One animal died because of untreatable ventricular fibrillation, resulting in a final study cohort of 14 animals. Downstream experiments included less animals at timepoints due to different timepoint sampling. All animals were intact females with body weights (BW) of 75–85 kg. To guarantee the absence of infectious diseases with a possible impact on the cardiovascular system, standard vaccination strategies against *Haemophilus parasuis*, porcine circovirus 2, porcine parvovirus, *Erysipelothrix rhusiopathiae*, and *Escherichia coli* were implemented by the breeder. Upon arrival at our facility, all animals were visually examined regarding their general behavior, posture and gait, skin color, breathing pattern, appetite, and fecal and urinary excretion. If abnormalities were observed, a physical examination was additionally conducted. Each animal was guaranteed a minimum acclimatization period of 7 days before the experiment.

On the day of the experiment, animals were sedated with an intramuscular injection of ketamine (Ketasol^®^-100 ad us.vet.; Dr. E. Graeub AG, Berne, Switzerland; 15 mg/kg BW), azaperone (Stresnil^®^ ad us.vet.; Elanco Tiergesundheit AG, Basel, Switzerland; 2 mg/kg BW), and atropine (Atropine Sulfate KA 1%; Kantonsapotheke, Zürich, Switzerland; 0.05 mg/kg BW). Anesthesia was then induced by administration of propofol (Propofol^®^ Lipuro 1%; B. Braun Medical AG, Sempach, Switzerland; 1–2 mg /kg BW) in an intravenous line introduced in the V. auricularis until the swallowing reflex was repressed and intubation was possible. Anesthesia was maintained with a combination of constant intravenous infusion of propofol (Propofol^®^ Lipuro 2%; B. Braun Medical AG, Sempach, Switzerland; 3 mg/kg BW/h) and inhalation of isoflurane (Attane™ Isoflurane ad.us.vet.; Piramal Enterpr. India; 1.0–1.5%) in 70% oxygen:air mixture using mechanical ventilation. Insertion of a urinary catheter and monitoring urine production as well as performing repeated blood gas analysis allowed conclusions on fluid loss during anesthesia, which was compensated by constant infusion of Ringer’s solution (Ringerfundin^®^; B. Braun Medical AG, Sempach, Switzerland; 5–10 mL/kg BW/h). For pain relief, all pigs received intravenous injections of buprenorphine (Temgesic^®^; Indivior Schweiz AG, Baar, Switzerland; 0.01 mg/kg BW) every 5 h throughout the course of the experiment. Animals premedicated with the antiarrhythmic drug amiodarone (Cordarone; Sanofi-Aventis (Suisse) SA, Vernier, Switzerland; 1.5 mg/mL 5% glucose, slow dripping) at least 1 h prior to surgical intervention and effect levels were maintained until the end of the experiment. Three ECG leads were placed in the following manner: one to the right of the sternum in the third intercostal space, one to the left of the sternum in the third intercostal space, and one to the left at the costochondral junction in the fourth intercostal space. These locations correspond to the anatomical positions of the right ventricle, the interventricular septum, and the left ventricle. Invasive blood pressure measurements were performed through a central arterial line.

### Experimental setup and timeline

Sixty minute following carotid crush maneuver, animals were subjected to either microthrombi injection (MINOCA) or a 90-min balloon occlusion with subsequent reperfusion (MI). In parallel, plasma and blood samples were collected prior to, 150 min and 300 min after infarction induction. After 300 min, animals were euthanized, and MRI was performed to assess infarction areas followed by tissue sampling.

### Microthrombus generation and MINOCA induction

A midline incision of approximately 15 cm was performed on the ventral side of the neck with the animal in dorsal recumbence. The right carotid artery was bluntly dissected over a section of approximately 10 cm. Using a standard hemostatic clamp, the entire distal half of the prepared carotid artery was crushed by clamping a standard hemostatic clamp and holding for 5 s, before repeating adjacently. Proximal and distal ligatures were used to slightly reduce blood flow during thrombus formation and thus prevent thrombus flushing. Thirty minutes post-crush, the carotid artery was examined for the progress of thrombus formation by direct supravascular ultrasound, and a second series of crushes was performed as described above if no thrombus formation was detected. Sixty minutes following carotid crush, the carotid artery was ligated and excised. The thrombus material formed within the carotid artery was shredded and filtered through multiple pore-sized filters to create 200 microthrombi with a size of approximately 150–200 µm, which were injected to induce MINOCA as described previously [8].

The right femoral artery was catheterized with an introducer sheath (Avanti^®^ + Introducer, 10F; Cordis^®^, Miami Lakes FL, USA) placed percutaneously under ultrasound guidance using the Seldinger method. Heparin was administered (Na-Heparin; B. Braun Medical AG, Sempach, Switzerland) until an Activated Clotting Time of > 250 s was reached. A 7F guiding catheter (Medtronic Launcher, Minneapolis, MN, USA, 7F, AL 1.0, 90 cm), introduced over the right femoral arterial access, was used to engage the left coronary artery and place a coronary guide wire (Abbott, Santa Clara, CA, USA, Balance middleweight, 0.014″ × 190 cm) in the LAD artery.

Subsequently, a thru-lumen balloon catheter was introduced over the wire and positioned in the mid/proximal LAD. MT was injected through the balloon catheter with the balloon inflated to avoid acute backflush of the MT. Total balloon occlusion time resulted in approximately 5 min.

Following MT injection, a control angiography was performed by injection of 10 ml contrast agent (Ultravist^®^-300, Bayer Vital GmbH, Leverkusen, Germany) through the guiding catheter, all catheters were removed, and anticoagulation of the animal was stopped for the ACT to return to baseline. Angiographic assessment of coronary flow was repeated until 300 min post-MT injection.

### MI induction

As mentioned earlier in microthrombus generation and MINOCA Induction, the same carotid crush timeline and procedure were conducted.

The right femoral artery was catheterized with an introducer sheath (Avanti^®^ + Introducer, 10F; Cordis^®^, Miami Lakes FL, USA) and placed percutaneously under ultrasound guidance using the Seldinger method. Heparin was administered to the animal (Na-Heparin; B. Braun Medical AG, Sempach, Switzerland) until an activated clotting time of > 250 was reached. A 7F guiding catheter (Medtronic Launcher, Minneapolis, MN, USA, 7F, AL 1.0, 90 cm) introduced over the right femoral arterial access was used to engage the left coronary artery and place a coronary guide wire (Abbott, Santa Clara, CA, USA, Balance middleweight, 0.014″ × 190 cm) in the LAD artery.

Subsequently, a thru-lumen balloon catheter was introduced over the guide wire and positioned in the LAD distal to the 1st diagonal branch. The balloon was subsequently inflated with a total occlusion time of 90 min and consecutive reperfusion.

### Cardiovascular magnetic resonance imaging (CMR)

Magnetic resonance imaging was performed on a clinical 1.5 T system (Achieva, Philips Healthcare, Best, the Netherlands), equipped with a five-channel cardiac receiver array. Post-mortem LGE measurements were performed upon euthanasia of the animal 20 min after injection of gadolinium-based contrast agent (0.2 mmol/kg b.w., Gadovist, Bayer, Germany). The sequence consisted of a 3D segmented inversion recovery gradient echo sequence with the following parameters: TE/TR of 2.2/4.6 ms, spatial resolution 0.75 × 0.75 × 2 mm^3^, parallel imaging (SENSE) reduction factor 2.2, with approximately 90 slices covering the left ventricle from apex to base in short-axis orientation and approximately 65 slices in four-chamber orientation. The optimal inversion delay was visually estimated using an inversion delay scout sequence, maximizing signal contrast between affected and remote myocardium (approximately 290 ms). Images were analyzed using Segment (Medviso AB, Lund, Sweden) to delineate hyper-enhanced areas. The enhanced myocardial volume is reported as a percentage of the total myocardial volume.

### Immunohistochemistry

After formalin fixation for at least 24 h at room temperature, the biopsies were dehydrated and embedded in paraffin. The prepared sections with a thickness of 3 μm were stained with hematoxylin (Artechemis, Zofingen, Switzerland) and eosin (Waldeck, Germany), and then digitized with a slide scanner (Zeiss Axio Scan.Z1, Carl Zeiss AG, Feldbach, Schweiz). Imaging was performed with equipment maintained by the Center for Microscopy and Image Analysis (ZMB), University of Zürich (Switzerland). Digital images of representative fields were captured, and analyses were performed using QuPath software (Version 0.5.1.) [3]. Hereby, necrotic regions, characterized by the loss of nuclei, cytoplasmic eosinophilia, and disruption of tissue architecture, were identified and quantified via hematoxylin and eosin (H&E) staining according to the Canadian Cardiovascular Society’s guidelines [33, 34], with 15–35 sections analyzed per animal. Interstitial bleeding was analyzed by determining regions displaying erythrocyte extravasation via H&E staining and quantified according to the Canadian Cardiovascular Society’s guidelines [33, 34], with 15–35 sections analyzed per animal. Finally, leukocyte infiltrate was quantified in sections from MI and MINOCA animals by analyzing the total number of cells in the MI and MINOCA infarcted zones and comparing them to the healthy zones of their respective models, with 5 sections per zone per animal, with one zone analyzed per animal. Within MINOCA animals, further immune cell infiltrate analyses were conducted by evaluating the number of immune cells in zones with an identifiable microthrombus (200 μm Feret diameter, the same size that were injected), the perivascular area with the thrombus and the surrounding vessel (maximum 400 μm Feret diameter), and the thrombus-associated zone (up to 1 mm from the thrombus) with 2–6 sections analyzed per animal. Determining the thrombus zone utilized similar metrics that have been used to determine the border zone via immunohistochemistry [59] and validated with existing spatial transcriptomic data that demonstrates the border zone is hundreds of micrometers thick [7]. Individual values per section were then averaged to have a mean value per animal for all immunohistochemistry analyses.

### miRNA isolation and miRNA profiling

MiRNA was extracted with the miRNeasy plasma advanced kit (Qiagen AG, Hombrechtikon, Switzerland), according to the manufacturer’s protocols. Briefly, 600 µL of plasma was loaded onto the RNeasy UCP MinElute spin column. After the appropriate washing steps, the miRNA was eluted with 20 µl RNase-free water. MiRNA sequencing libraries were prepared using QIAseq^®^ miRNA UDI Library Prep kit (Qiagen, Cat. No. 331905) according to the manufacturer’s instructions. As recommended for plasma samples, 5 μL of the miRNA eluate was taken as input material. 36 libraries were equimolar pooled and sequenced on 0.5 lane of NovaSeq 6000 S4 PE100 flowcell, aiming at ~ 20 million paired reads per library. Sequences of mature and hairpin microRNAs were downloaded from the miRBase database9. For microRNA quantification, the program quantifier.pl from the miRDeep2 package10 was used on the short reads in FASTQ format. Only quantification results for Sus scrofa microRNAs were used in downstream analysis.

### Multiplex cytokine ELISA

Concentrations of soluble molecules in plasma were quantified using the Luminex-based multiplex technology and the Milliplex Map Porcine Cytokine/Chemokine Magnetic Bead Panel comprising the most relevant soluble immune mediators (Merck Millipore, PCYTMG-23K-13PX, Burlington, USA) as described in manufacturer’s instructions. All samples were diluted 1 + 1 with sample diluent provided with the kit. Standards were reconstituted and prepared as described in the manufacturer’s instructions.

### Plasma and proteomic analysis

2 µL plasma of each sample was added to 45 µL of lysis buffer (4% Sodium dodecyl sulfate (SDS) in 100 mM Tris/HCl pH 8.2, supplier). The samples were boiled at 95 °C for 10 min while shaking at 800 rpm on a Thermoshaker (Eppendorf). Proteins were reduced with 2 mM TCEP (tris(2-carboxyethyl)phosphine) and alkylated with 15 mM chloroacetamide (Thermo Fisher) at 30 °C for 30 min.

Samples were processed using the single‐pot solid‐phase enhanced sample preparation (SP3). The SP3 protein purification, digest and peptide clean-up were performed using a KingFisher Flex System (Thermo Fisher Scientific) and carboxylate-modified magnetic particles (GE Life Sciences; GE65152105050250, GE45152105050250) [28]. Beads were conditioned following the manufacturer’s instructions, consisting of 3 washes with water at a concentration of 1 µg/µL. Samples were diluted with 100% ethanol to a final concentration of 50% ethanol. The beads, wash solutions and samples were loaded into 96 deep well- or micro-plates (Thermo Fisher) and transferred to the KingFisher. Following steps were carried out on the robot: collection of beads from the last wash, protein binding to beads, washing of beads in wash solutions 1–3 (80% ethanol), protein digestion (overnight at 37 °C with a trypsin:protein ratio of 1:50 in 50 mM Triethylammonium Bicarbonate (TEAB, Thermo Fisher) and peptide elution from the magnetic beads using MilliQ water (Merck). The digest solution and water elution were combined and dried to completeness and re-solubilized in 20 µL of MS sample buffer (3% acetonitrile, 0.1% formic acid, both from Thermo Fisher). Peptide concentration was determined using the Lunatic UV/Vis polychromatic spectrophotometer (Unchained Labs).

LC–MS/MS analysis was performed on an Orbitrap Fusion Lumos (Thermo Scientific) equipped with a Digital PicoView source (New Objective) and coupled to an M-Class UPLC (Waters). Solvent composition of the two channels was 0.1% formic acid for channel A and 99.9% acetonitrile in 0.1% formic acid for channel B. Column temperature was 50 °C. For each sample, 300 ng of peptides was loaded on a commercial ACQUITY UPLC M-Class Symmetry C18 Trap Column (100 Å, 5 µm, 180 µm × 20 mm, Waters) connected to a ACQUITY UPLC M-Class HSS T3 Column (100 Å, 1.8 µm, 75 µm × 250 mm, Waters). The peptides were eluted at a flow rate of 300 nL/min. After a 3-min initial hold at 5% B, a gradient from 5 to 22% B in 80 min and 22 to 32% B in additional 10 min was applied. The column was cleaned after the run by increasing to 95% B and holding 95% B for 10 min prior to re-establishing loading condition.

Samples were measured in randomized order. The mass spectrometer was operated in data-dependent mode (DDA) with a maximum cycle time of 3 s, funnel RF level at 40%, heated capillary temperature at 275 °C, and Advanced Peak Determination (APD) on. Full-scan MS spectra (300 − 1′500 m/z) were acquired at a resolution of 120,000 at 200 m/z after accumulation to an automated gain control (AGC) target value of 500,000 or for a maximum injection time of 40 ms. Precursors with an intensity above 5000 were selected for MS/MS. Ions were isolated using a quadrupole mass filter with 0.8 m/z isolation window and fragmented by higher energy collisional dissociation (HCD) using a normalized collision energy of 35%. Fragments were detected in the linear ion trap with the scan rate set to rapid, the automatic gain control set to 10,000 ions, and the maximum injection time set to 50 ms. Charge state screening was enabled, and singly, unassigned charge states and charge states higher than seven were excluded. Precursor masses previously selected for MS/MS measurement were excluded from further selection for 20 s, applying a mass tolerance of 10 ppm. The samples were acquired using internal lock mass calibration on m/z 371.1012 and 445.1200.

The acquired MS raw data were processed for identification and quantification using FragPipe (version 17.0), MSFragger (version 3.4) and Philosopher (version 4.5.1). Spectra were searched against Uniprot Sus scrofa reference proteome (UP000008227, canonical version from 2022-07-25), concatenated to its reversed decoyed fasta database and common protein contaminants. For the closed search settings, strict trypsin digestion with a maximum of 2 missed cleavages was set. Carbamidomethylation of cysteine was set as fixed modification, while methionine oxidation and N-terminal protein acetylation were set as variable. Label-free quantification and match between run option were enabled.

### Single-ELISA

Levels of leukotriene B4 in undiluted plasma were assessed using the Leukotriene B4 Multispecies Competitive ELISA Kit (Invitrogen) according to manufacturer’s instruction. For tissue analysis, tissue was extracted from healthy and infarcted regions identified by MRI and visual inspection and stored in RNAlater. For cell lysis, tissue was thawed and weight was determined. 15–20 mg of tissue was incubated in modified RIPA buffer for 10 min at 4 °C. Tissue was homogenized 4 times at maximum speed for 20 s. Homogenates were incubated for an additional 30 min at 4 °C under orbital shaking. Tissue lysates were centrifuged and LTB4 concentration was measured using the mentioned ELISA. Concentrations were normalized to 15 g of tissue.

### PBMC isolation and stimulation assay

PBMCs were isolated from full blood samples via density gradient centrifugation using SepMate (Stemcell) tubes and lymphoprep (Lymphoprep) density gradient medium. Briefly, blood was diluted 1:1 with PBS, and added to the SepMate Tubes containing lymphoprep density gradient medium. Tubes were then centrifuged at 1200 g for 10 min and the top layer containing enriched PBMCs was poured off into a new tube. Subsequently, PBMCs were washed twice with PBS. 1–1.5 × 10^6^ PBMC were then plated in a 96-well plate in 500 µl RPMI (Gibco) with GlutaMax (Gibco) supplemented with 100 µg/ml streptomycin, 100 U/ml penicillin for 24 h at 37 °C, 5% CO_2_. Subsequently, cells were stimulated with 1 μg/mL LPS (Sigma) und 100 nM LTB4 (Sigma) for 8 h. Supernatants were collected and subsequently analyzed for TNF-α expression using ELISA (Invitrogen, KSC3011).

### Statistical analysis

Kolmogorov–Smirnov and d'Agostino and Pearson omnibus normality tests were applied to verify Gaussian distribution. Troponin T and Creatine Kinase data were compared using a repeated measures two-way ANOVA with a Geisser–Greenhouse correction, alongside an uncorrected Fisher’s least significant difference test. All functional data were compared using a student’s T-test. All histology data were compared using either via a Brown–Forsythe and Welch ANOVA with a Dunnett’s T3 multiple comparisons test or a two-tailed unpaired t-test with Welch’s correction for proving statistical significance. Plasma ELISA data were compared using a repeated measure 2-way ANOVA (mixed model) with a Geisser–Greenhouse correction and Tukey’s post hoc correction for multiple comparisons. Tissue ELISA was compared using a Brown–Forsythe and Welch ANOVA with a Dunnett’s T3 multiple comparisons test. Finally, cell data were compared using a two-tailed unpaired *t*-test with Welch’s correction between individual conditions at 0 min vs. 300 min. The level of significance was determined to be *p* < 0.05 (GraphPadPrism V8, La Jolla, CA, USA). Proteomic data were analyzed using DIANN, with significance accepted with FDR ≤ 0.1. miRNA data were analyzed in R through DESeq2, with significance accepted with FDR ≤ 0.1. All statistical analyses were performed in GraphPad Prism V8.

## Results

### MINOCA and MI produce significantly different infarct sizes

**Fig. 2:**
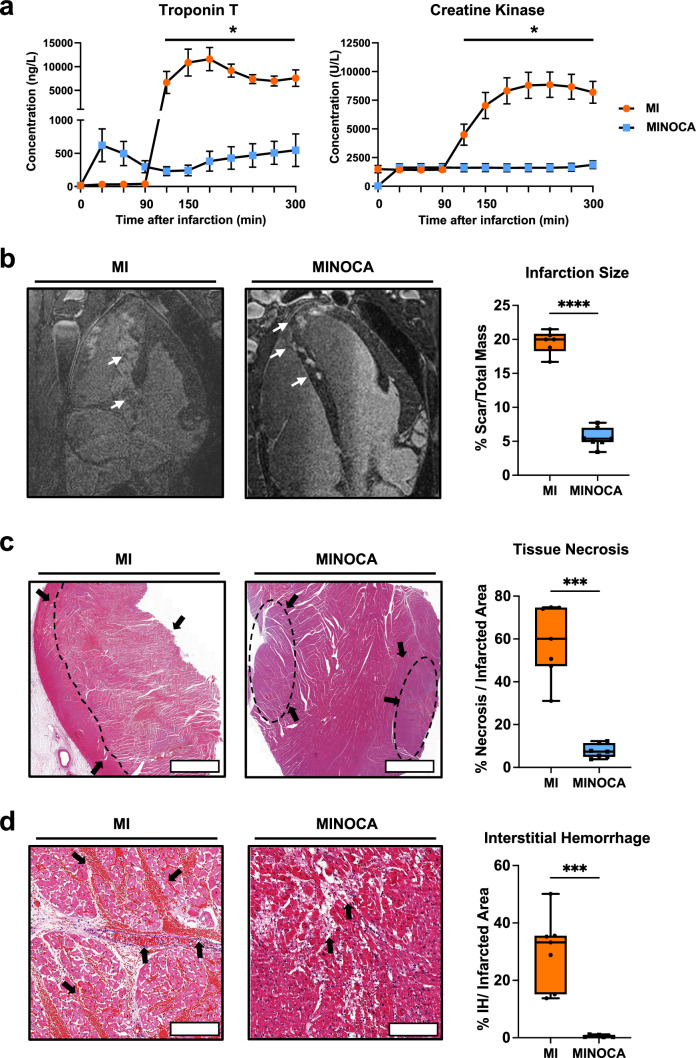
CME-derived MINOCA and MI display significantly different infarction sizes accompanied by cell death and interstitial hemorrhage.** a **Troponin and CK kinetics following infarction induction in both, MI and MINOCA animals. N = 7 for MI and MINOCA. **b** Percentage of scar tissue of total ventricular mass as calculated by ex-vivo LGE CMR in both, MI and MINOCA animals. White arrows in MRI images indicate areas of infarction. N = 6 for MI, N = 7 for MINOCA. **c** Percent tissue necrosis in AHA heart segments affected either by MI or MINOCA, as evaluated by histology of tissues in the infarction area. Scale bar = 4 mm. N = 7 for MI and MINOCA. **d** Frequency of interstitial hemorrhage in the infarction area as assessed by H&E in the infarction area of either MI or MINOCA animals. N = 7 for MI and MINOCA. Arrows denote specific areas of interest. Data are displayed as box and whisker plots with individual points. Boxplots display the 95% confidence interval. Scale bar = 400 µm. Statistical significance for (**a**) was determined using a two-way ANOVA comparing the two models at their respective timepoints. Statistical significance for (**b**), (**c**), and (**d**) was determined by a two-tailed unpaired T-test with Welch’s correction. Asterisks indicate *p* values: *p ≤ 0.05, ***p ≤ 0.001, ****p ≤ 0.0001, only significant values are shown. Abbreviations: MI, classic myocardial infarction; MINOCA, myocardial infarction with non-obstructive coronary arteries; LGE, Late Gadolinium Enhancement; CMR, cardiovascular magnetic resonance

We first evaluated cardiac markers in both MINOCA and MI pigs to confirm key differences already present in clinical data. Following reperfusion (90 min after balloon occlusion) and up to 300 min post-reperfusion, animals subjected to MI displayed significantly higher levels of troponin (maximum level: 7580.7 ± 4292.3 ng/mL), while MINOCA animals reached a maximum troponin level of 547.0 ± 489.2 ng/mL (Fig. 2a). Similarly, creatine kinase (CK) was found to be much higher in MI animals (7580.7 ± 4292.3 U/L), while MINOCA animals had maximal CK levels of 1827.8 ± 677.3 U/L (Fig. 2a). We also determined the hemodynamic parameters (systolic arterial pressure, diastolic arterial pressure, mean arterial pressure, and heart rate) for both MINOCA and MI animals that completed the study, demonstrating no differences between both groups across the duration of the experimental timeline (Table 1) alongside no adverse effects per animal (Supplementary Table 1).

**Table 1:** Mean hemodynamic parameters for MINOCA and MI animals

Groups	Baseline	45 min of occlusion	Post microthrombi injection/reperfusion					
			0 min	60 min	120 min	180 min	240 min	300 min
*Systolic arterial pressure (SAP; mmHg)*								
MINOCA	106 ± 11	n/a	93 ± 17	104 ± 3	99 ± 4	97 ± 3	93 ± 4	93 ± 7
MI	105 ± 14	94 ± 17	94 ± 16	91 ± 12	87 ± 14	82 ± 11	75 ± 14	83 ± 10
*Diastolic arterial pressure (DAP; mmHg)*								
MINOCA	67 ± 14	n/a	59 ± 10	63 ± 3	62 ± 5	57 ± 4	54 ± 2	66 ± 13
MI	62 ± 10	61 ± 12	60 ± 9	53 ± 8	48 ± 9	44 ± 7	44 ± 9	48 ± 9
*Mean arterial pressure (MAP; mmHg)*								
MINOCA	88 ± 10	n/a	77 ± 12	84 ± 3	80 ± 5	77 ± 3	73 ± 3	79 ± 8
MI	84 ± 11	78 ± 14	77 ± 12	72 ± 9	67 ± 10	63 ± 9	60 ± 11	66 ± 9
*Heart rate (HR; bpm)*								
MINOCA	69 ± 10	n/a	68 ± 7	73 ± 14	74 ± 9	74 ± 11	76 ± 9	75 ± 16
MI	63 ± 6	67 ± 14	66 ± 13	64 ± 9	66 ± 5	69 ± 4	72 ± 9	75 ± 12

Myocardial ischemia reperfusion injury is known to exert a significant inflammatory response associated with augmented tissue injury, infarct expansion and compromised cardiac function following MI [16]. For MINOCA, a significant inflammatory response arising from CME has been suggested to be causal for subsequent decline in myocardial function despite smaller infarction areas when compared to MI [11, 36]. Thus, we measured the overall infarction areas in both MINOCA and MI animals (Fig. 2b).

Late gadolinium enhancement MRI determined patchy infarction areas in MINOCA animals (2.3 ± 0.8% of total ventricular mass), which was significantly less compared to MI animals (19.5 ± 1.5%). While we previously found that MINOCA animals exhibited an ejection fraction of 60 ± 13% [8], MI animals averaged an ejection fraction around 58.6 ± 1.9% (Supplementary Table 2). Consistently, histological assessment of affected tissues revealed significantly less areas of necrosis in myocardial areas of infarction following MINOCA compared to MI (Fig. 2c). In addition, it revealed that MINOCA had nearly nonexistent bleeding, while MI animals displayed significant interstitial bleeding in infarcted areas (Fig. 2d).

### MINOCA and MI display significantly different inflammatory cell infiltration patterns in the infarction area

**Fig. 3:**
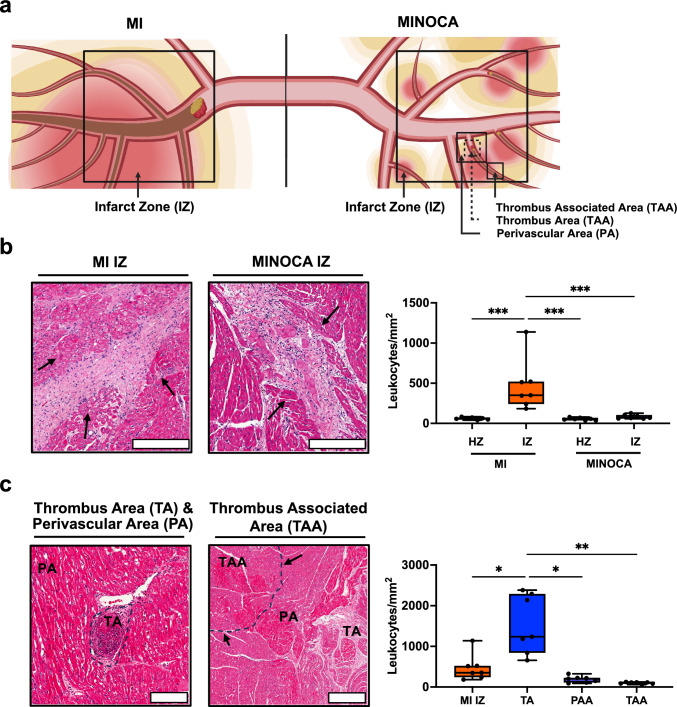
MINOCA and MI display significantly different inflammatory cell infiltration patterns in the infarction area. **a** Graphical overview on different sections assessed during histology via H&E staining. **b** Leukocyte infiltrates in the supply area of the coronary artery undergoing either balloon occlusion (MI) or microthrombus injection (MINOCA) were quantified in tissue samples. Arrows mark leukocyte infiltrates. Scale bar: 400 µm. Statistical significance was determined via a Brown–Forsythe and Welch ANOVA with a Dunnett’s T3 multiple comparisons test. **c** Leukocyte infiltrates in specific areas affected by microthrombosis were quantified in various regions of MINOCA animals, starting with the thrombus area (TA), the perivascular area (PA), and the thrombus-associated area (TAA). Arrows denote specific areas of interest. Scale bar for TA and PA: 200 µm. Scale bar for TAA: 400 µm. Data are displayed as box and whisker plots with individual points. Boxplots display the 95% confidence interval. Statistical significance was determined via a Brown–Forsythe and Welch ANOVA with a Dunnett’s T3 multiple comparisons test. Asterisks indicate *p* values: **p ≤ 0.01, ***p ≤ 0.001, ****p ≤ 0.0001, only significant values are shown. Abbreviations: MI, classic myocardial infarction; MINOCA, myocardial infarction with non-obstructive coronary arteries; H&E, hematoxylin and eosin; HZ, healthy zone; IZ, infarction zone; TA, thrombus area; PA, perivascular area; TAA, thrombus-associated area

Motivated by the PLATO trial demonstrating higher inflammatory response in MINOCA patients, we then investigated whether immune cells migrate over to areas of ischemic injury in MINOCA animals. Thus, we quantified inflammatory cell infiltrates in tissue samples via hematoxylin and eosin staining (H&E) from myocardial supply areas of the coronary artery subjected to either microthrombi injection or balloon occlusion (Fig. 3a). Immune cell infiltrate is a key paradigm in MI. Through histological measurements, we determined that the global MINOCA immune cell infiltrate in the infarct zone was significantly less than the infiltrate in the MI-infarcted zone (Fig. 3b).

In MINOCA animals, we also measured the immune cell infiltrate present at varying distances from the microthrombus location (Fig. 3a). We defined three zones respective to visualizing leukocyte infiltrate: the thrombus area (TA), the perivascular area (PA), and the thrombus-associated area (TAA). Comparing these defined zones to the immune cell infiltrate in MI animals, we measured a higher amount of immune cell infiltrate present in the MINOCA thrombus zone compared to the PA and TAA, demonstrating greater infiltration at the TA (Fig. 3c). Even when compared to the MI infiltrate, there is higher inflammation present in the thrombus area, demonstrating that the more profound inflammation in MINOCA may be due to the increased immune cell infiltrate present at the thrombus site (Fig. 3c). Thus, while the global MINOCA immune cell infiltrate is less than in MI, thrombotic areas elicit higher inflammation than in MI by concentrating inflammatory signaling at those sites.

### MINOCA and MI promote significant inflammatory responses with distinct lead cytokines

**Fig. 4:**
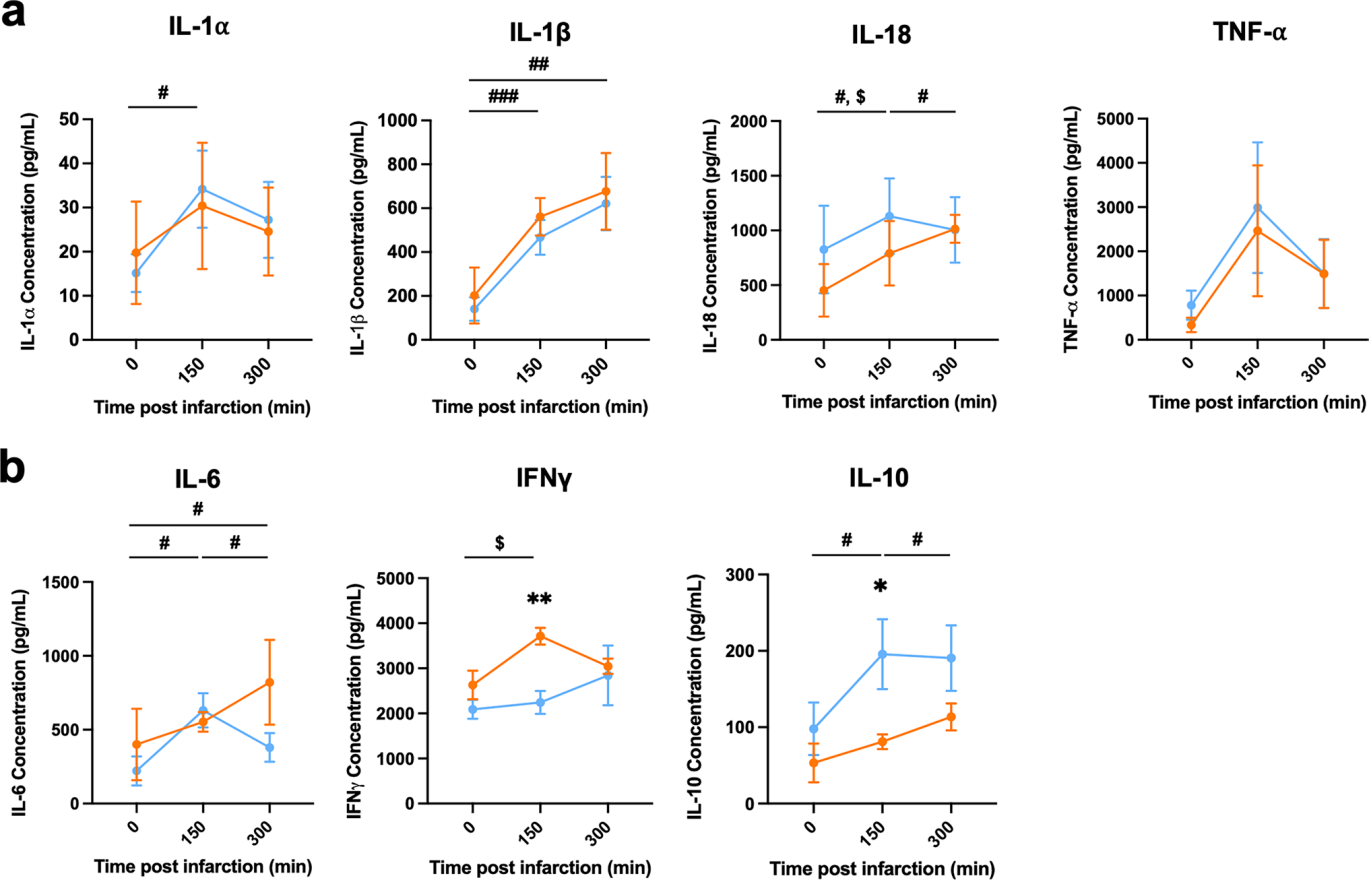
MINOCA and MI induce prominent inflammatory responses with lead cytokine differences. Serum samples were collected 0, 150, and 300 min after infarction induction in both MI (red) and MINOCA (blue) animals were collected and subsequently pro-inflammatory and anti-inflammatory cytokine expression were assessed using multi-ELISA. **a** Cytokines with similar temporal kinetics of both, MI and MINOCA animals.** b** Significantly different cytokines in samples of both, MI and MINOCA animals across sampling timepoints. Data are displayed as mean ± SEM. Asterisks indicate *p*-values: *p ≤ 0.05, **p ≤ 0.01 (comparison of disease models at a given timepoint); #*p* ≤ 0.05, ##*p* ≤ 0.01, ###*p* ≤ 0.001 (comparison of MINOCA across timepoints); $*p* ≤ 0.05 (comparison of MI across timepoints) with only significant values shown. N = 4 for MI and N = 6 for MINOCA. Abbreviations: MI, classic myocardial infarction; MINOCA, myocardial infarction with non-obstructive coronary arteries

Myocardial injury is also canonically known to elicit significant inflammatory responses in MI, with a high pro-inflammatory, innate immune cell response [6]. Given the finding of higher inflammation within the TA zone in MINOCA animals, we thus dissected the early systemic inflammatory response following MINOCA by evaluating lead cytokines relative to MI. We performed a multi-ELISA analysis to quantify the most relevant soluble immune mediators up to 300 min post-MINOCA and MI induction. Although infarction sizes were significantly smaller in MINOCA animals, plasma levels of pro-inflammatory, innate immune cytokines monitored including tumor necrosis factor-alpha (TNF-α) as well as interleukin-1α (IL-1α) and IL-1β, which are crucial inflammatory mediators of myocardial ischemia reperfusion injury (IRI), were equally increased over time in both groups (Fig. 4a). Of note, IL-1α and IL-1β were found to be differentially expressed in MINOCA animals over time, with IL-18 peaking in MI animals at 150 min. In comparison, more canonically known anti-inflammatory cytokines, IL-1ra and IL-4 were found to be higher in MINOCA over time (Supp. Fig. 1a). However, the anti-inflammatory cytokine IL-12 (Supp. Fig. 1a) alongside the T-cell activating cytokine IL-2 (Supp. Fig. 1b) were not found to be statistically different up to 300 min post-MI or MINOCA induction. Uniquely, MI animals displayed augmented levels of interferon-gamma (INF-γ) relative to MINOCA across time, while MINOCA animals exhibited a different kinetic of the anti-inflammatory cytokine IL-10 (Fig. 4b). When compared to MI, these results indicate a distinct inflammatory response instigated through MINOCA, particularly via IL-10 activation, while MI had higher INF-γ.

### MINOCA and MI display distinct miRNA profiles linked to ischemia reperfusion injury

**Fig. 5:**
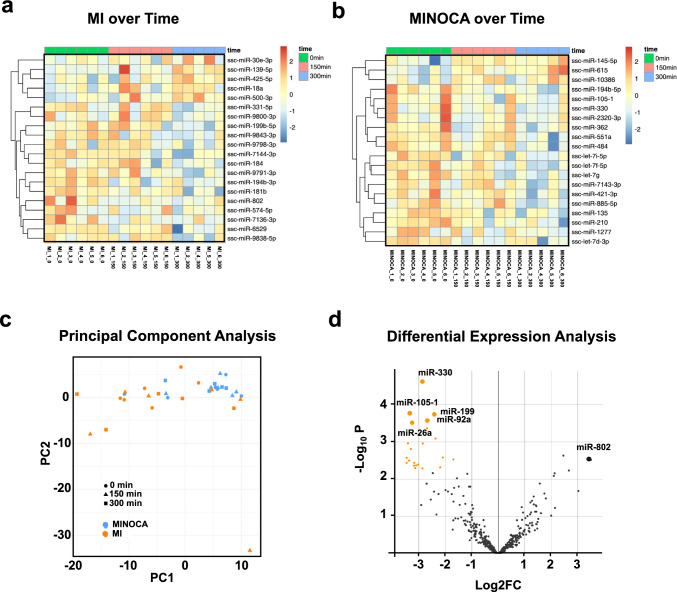
CME-derived MINOCA and MI induce distinct miRNA profiles of ischemia reperfusion injury. miRNA of plasma samples at 0, 150, and 300 min following infarction induction in both MI and MINOCA animals was isolated. Subsequently, miRNA profiling was performed.** a** Heatmap of altered miRNA expression levels in MI animals over time.** b** Heatmap of altered miRNA expression levels in MINOCA animals over time.** c** Principal component analysis of miRNA profiles in MI and MINOCA animals over time. miRNAs with highest FDR and fold change are highlighted for both MI and MINOCA animals. **d** Differential expression analysis comparing MI and MINOCA depicted in a volcano plot. Significantly different expressed miRNAs are colored. N = 6 for MI and MINOCA plasma taken at 0 min and 150 min. N = 5 for MI and MINOCA plasma taken at 300 min. Statistical significance was determined by FDR ≤ 0.1, Log2FC ≥ 1.5, and Log2FC ≤ − 1.5. Abbreviations: MI, classic myocardial infarction; MINOCA, myocardial infarction with non-obstructive coronary arteries

In recent literature, microRNAs (miRNAs) have also been implicated in driving cardiac injury and inflammation following MI and CME-derived tissue injury and inflammation [20, 60]. Thus, miRNA expression represented a differentiating category in finding unique MINOCA biomarkers. To find other biomarkers that may differentiate MINOCA from MI, we isolated miRNA from plasma samples to perform miRNA transcriptome sequencing. After 300 min post-MI induction, the MI group displayed an upregulation of miRNAs linked to myocardial ischemia and reperfusion including ssc-miR-139-5p, ssc-miR-18a [38] as well as ssc-miR-30[61] (Fig. 5a). MINOCA in turn induced a more heterogeneous miRNA profile with altered expression of ssc-miR-615 and ssc-miR-484 and downregulation of ssc-miR-145-5p (Fig. 5b). A principal component analysis revealed clustering of miRNA profiles of animals undergoing MI and MINOCA over time (Fig. 5c). Moreover, when comparing the systemic miRNA profiles of MINOCA and MI in a differential expression analysis, a distinct miRNA profile with upregulation of miRNAs associated with cardiac IRI and cardiomyocyte death was observed (Fig. 5d). In the plasma of animals undergoing MI, ssc-miR-330, ssc-miR-105, ssc-miR^−^26a, ssc-miR-199-3p, and ssc-miR-92a all related to cardiac IRI were significantly upregulated. In MINOCA animals in turn, ssc-miR-802 had been upregulated when compared to those of MI counterparts, however not reaching statistical significance (Fig. 5d). These data indicate that CME-induced MINOCA has a distinct molecular pattern of cardiac injury, particularly in mediating the inflammatory responses occurring in MINOCA when compared to coronary occlusion/reperfusion induced MI. Such molecular patterns may serve to discriminate both disease pathologies.

### Leukotriene signaling displays a distinct inflammatory signature of MINOCA

**Fig. 6:**
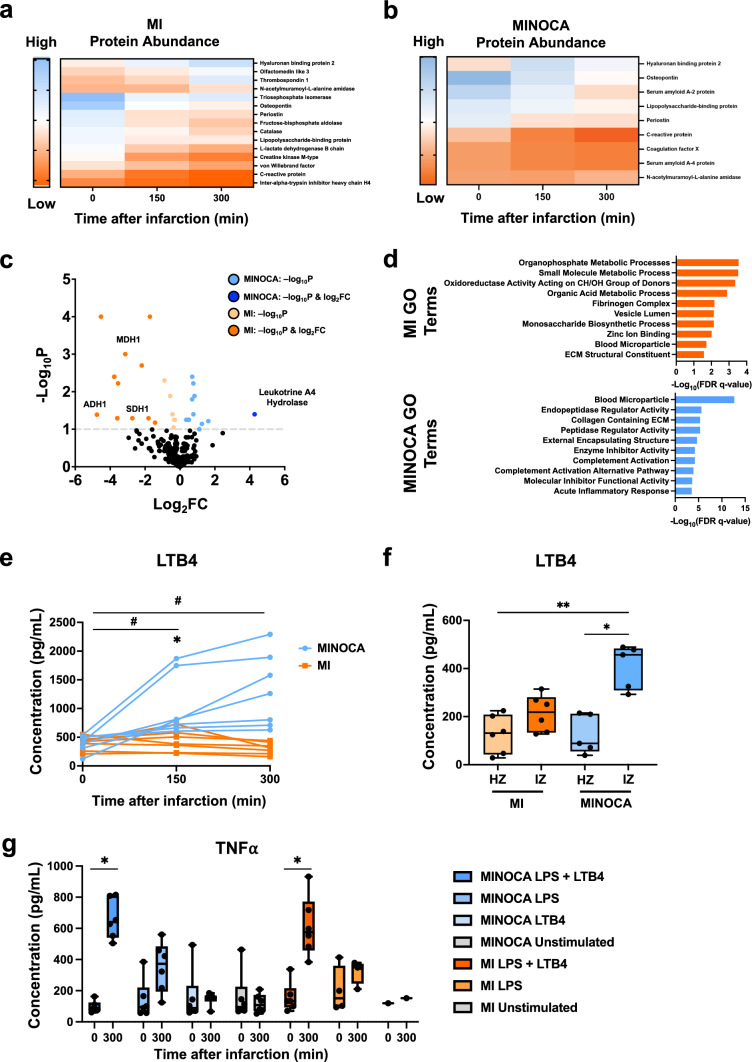
Leukotriene signaling constitutes a inflammatory pathway underlying MINOCA. **a**–**d** Plasma samples were collected at 0, 150, and 300 min following infarction induction in both MI and MINOCA animals. Subsequently, a proteomic analysis was performed.** a** Heatmap of significantly altered protein expression levels in MI animals over time. The heatmap scale bar represents the mean normalized protein abundance measured via LC/MS, with high mean normalized abundance in blue and low mean normalized abundance in orange.** b** Heatmap of significantly altered protein expression levels in MINOCA animals over time. The heatmap scale bar represents the mean normalized protein abundance measured via LC/MS, with high mean normalized abundance in blue and low mean normalized abundance in orange.** c** Differential expression analysis displaying significantly differently expressed proteins between MINOCA and MI at 300 min in a volcano plot. Proteins involved in anaerobic glycolysis are highlighted for MI animals, while leukotriene A4 hydrolase is highlighted for MINOCA animals. N = 6 for MINOCA and MI. Statistical significance was determined by FDR ≤ 0.1, Log2FC ≥ 1.5, and Log2FC ≤ -1.5. **d** Gene ontology enrichment via gene set enrichment analysis (GSEA) of differentially expressed proteins specific to MI (blue) and MINOCA (orange). Statistical significance was determined with a Kolmogorov–Smirnov test through the GSEA program, with FDR-q-value ≤ 0.05.** e** ELISA of LTB4 in plasma samples of MI and MINOCA animals over time. Data are displayed as a repeated measure graph with each replicate connected. Statistical significance was determined by a repeated measure 2-way ANOVA (mixed model) with a Geisser–Greenhouse correction and Tukey’s post hoc correction for multiple comparisons. Asterisks indicate *p* values: *p ≤ 0.05 (comparison of disease models at a given timepoint); and #*p* ≤ 0.05 (comparison of MINOCA across timepoints), with only significant values shown. **f** ELISA of LTB4 in tissue samples of infarcted (IZ) and healthy (HZ) myocardium of MINOCA and MI animals. Data are displayed as box and whisker plots with individual points. N = 5 for MINOCA, N = 6 for MI. **g** PBMCs isolated from animals either undergoing MINOCA or MI were subjected to stimulation with LTB4 and/or LPS. Subsequently, TNF-α expression was measured. Data are displayed as box and whisker plots with individual points. Boxplots display the 95% confidence interval. Statistical significance was determined by Student’s T-test. N = 4–6 for each condition, except the MI unstimulated control with N = 1. Abbreviations: MI, classic myocardial infarction; MINOCA, myocardial infarction with non-obstructive coronary arteries; HZ, healthy zone; IZ, infarction zone; PMBCs, peripheral mononuclear blood cells; LTB4, leukotriene B4; LPS, lipopolysaccharide. Statistical significance was determined via a Brown–Forsythe and Welch ANOVA with a Dunnett’s T3 multiple comparisons test. Asterisks indicate *p*-values: *p ≤ 0.05, **p ≤ 0.01. Abbreviations: MI, classic myocardial infarction; MINOCA, myocardial infarction with non-obstructive coronary arteries; HZ, healthy zone; IZ, infarction zone; PMBCs, peripheral mononuclear blood cells; LTB4, leukotriene B4; LPS, lipopolysaccharide

With the distinct inflammatory differences measured between MINOCA and MI, we then measured how the downstream protein expression changed within MINOCA pathophysiology. Here, we wanted to uncover novel markers that can further delineate pathways that are involved in the MINOCA inflammatory response. Thus, we performed a proteomic analysis of plasma samples collected at 150 min or 300 min following infarction induction. We observed a consecutive upregulation of molecules associated with inflammation in MI animals throughout the 300-min follow-up period, with strong upregulation of osteopontin, periostin and c-reactive protein (Fig. 6a). MINOCA animals displayed similar kinetics with progressive expression of osteopontin, periostin and c-reactive protein (Fig. 6b). However, proteins of the coagulation cascade had also been significantly upregulated in MINOCA animals. An upregulation of leukotriene hydrolase A4 (LTA4H) was detected in the plasma of MINOCA animals when compared to animals undergoing MI (Fig. 6c). To further validate our findings, we performed gene ontology (GO) enrichment of the differentially expressed proteins found in MINOCA or MI, where in MINOCA we measured acute inflammatory signaling (C3, ORM1, SERPINA1, SAA4*)* alongside complement signaling (C3, CFH, C5), while in MI we found metabolic processes (MDH2, PDH2), and the fibrinogen complex (FN1, SERPINF2, FGB) (Fig. 6d).

Notably, when quantifying systemic leukotriene B4 (LTB4) levels, the enzymatic product of LTA4H, in the plasma of MINOCA and MI throughout 300 min following infarction using ELISA, we also observed a significantly higher concentration of LTB4 in MINOCA animals compared to baseline (as indicated by #) at both 150 min and 300 min. We also quantified higher concentration of LTB4 at 300 min when compared to MI (as indicated by *) (Fig. 6e). Consistent with these observations, tissue samples collected from areas affected by CME in MINOCA animals displayed increased levels of LTB4 compared to the MINOCA healthy zone, indicating that CME-induced MINOCA may accelerate leukotriene signaling (Fig. 6f). Finally, we isolated peripheral mononuclear blood cells (PMBCs) from MINOCA and MI animals, where we subjected them to lipopolysaccharide (LPS) alone or with LTB4. Using TNF-α as a measurement of pro-inflammatory signaling, we determined that LTB4 addition in a pro-inflammatory environment augments pro-inflammation in both MINOCA and MI (Fig. 6g). Thus, at acute timepoints, we have further differentiated MINOCA from MI by showing MINOCA elicits higher dysregulated inflammatory signaling.

## Discussion

In this work, we have characterized a molecular pattern of CME-induced MINOCA which is distinct from MI through multiple modalities. We made use of a translational animal model mimicking all relevant clinical aspects of CME-derived MINOCA [8], which was aligned to the PLATO trial that delineated MI and MINOCA clinical data. As a result, we confirmed potential diagnostic biomarkers and therapeutic targets. Here, we demonstrated differences in infarct size, inflammatory responses, and differential miRNA and proteomic markers between MINOCA and MI. Based on the different and specific inflammatory induced responses, a clinically relevant discrimination of both infarction types may be possible to evaluate potential diagnostics and therapeutics for MINOCA.

The MINOCA and MI clinical data demonstrate difference in cardiac markers levels such as troponin, NT-pro-BNP, and GDF-15 levels [25, 26]. Our data comparing MINOCA to MI not only confirm the observed difference in plasma troponin levels, but also expand on further differences, such as higher plasma creatine kinase levels, infarct size, tissue necrosis, and interstitial bleeding in MI relative to MINOCA (Fig. 2). While we present functional data of both MI (Supplementary Table 2) and MINOCA animals [8], it is important to also acknowledge that some MI animals received catecholamine support during cMRI; hence, these values should be interpreted with caution. Moreover, we also assess overall interstitial hemorrhage using hematoxylin and eosin staining. This method allows for the quantitative evaluation of extravasated red blood cells in both MINOCA and MI samples. Hemorrhage is known to worsen inflammation and myocardial damage [19]. Thus, we incorporated hemorrhage into our evaluation of myocardial injury. With all these findings in mind, we were able to quantify the myocardial ischemia and reperfusion damage with respect to the Canadian Cardiovascular Society’s classification of acute myocardial infarction [33, 34]. Based on our findings, our MINOCA model closely mimics CCS Stage 3, cardiomyocyte necrosis and microvascular obstruction. In contrast, our MI model mimics CCS Stage 4, cardiomyocyte necrosis with microvasculature obstruction and bleeding [33, 34].

We also noted differentiating immune cell infiltration (Fig. 3), plasma-cytokines (Fig. 4), miRNA levels (Fig. 5), and proteomic (Fig. 6) differences that help differentiate MINOCA pathology. Moreover, instead of inert microspheres, our CME-derived MINOCA model utilizes autologous thrombus material, which induces a biological interplay with inflammatory as well as endothelial cells and adjacent cardiomyocytes. Hence, clinically relevant processes such as endogenous fibrinolysis can be activated, while the thromboembolic materials releases thrombogenic and pro-inflammatory factors with the potential to affect functional impairment of the coronary microcirculation and promote tissue inflammation [24, 32]. In addition to demonstrating key differences in our two porcine models, we also demonstrated that our preclinical data are consistent with and even expand clinical data to illustrate key inflammatory differences between MINOCA and MI.

While CME-induced MINOCA as well as MI demonstrated a largely analogous profile of plasma inflammatory cytokines (such as TNF-α, IL-1α and IL-1β) (Fig. 4a), differences in INF-γ (higher in MI) (Fig. 4b) and IL-10 (higher in MINOCA) (Fig. 4c) levels support the concept of distinct inflammatory responses, which require further investigation including the identification of relevant leukocyte subsets in both infarction types. The upregulation of these inflammatory cytokines is known to be crucial inflammatory mediators of myocardial ischemia reperfusion injury (IRI) and subsequent decline of cardiac function [17, 40, 43, 49]. Indeed, we show prominent inflammatory cell infiltrations in infarction areas exhibiting microthrombosis in animals undergoing MINOCA, which thus localize into the surrounding myocardium, acting as a “funnel” for inflammatory cells to further infiltrate into the tissue (Fig. 3). Consistently, significant cellular infiltrates of neutrophils and macrophages in microthrombi areas have also been detected in patients succumbing to CME [24, 32]. In contrast, animals undergoing MI exhibit rather dispersed inflammatory cell infiltrates throughout the entire area of infarction.

Here, we directly compared the miRNA profiles in MI and MINOCA at acute timescales. In the MI group over time, we were able to measure significant miRNAs associated with myocardial ischemia and reperfusion (Fig. 5). Particularly, ssc-miR-30 [61] is known to be associated with vascular intima injury following balloon twitching [61]. While we note that there were no differentially expressed miRNAs found in the MINOCA group (Fig. 5b), over time, there were markers of myocardial injury and promotion of cardiomyocyte apoptosis via upregulation of ssc-miR-615 [29] and ssc-miR-484 [39]. Of note, there was downregulation of ssc-miR-145-5p [56], which has been shown to protect the ischemic heart from inflammation in an MI model. With the highest fold change for MINOCA, ssc-miR-802 for instance, has been shown to promote cardiomyocyte apoptosis following MI through Sonic Hedgehog signaling (Fig. 5d) [37].

We identified leukotriene signaling with augmented systemic and local LTB4 levels as a specific molecular pathway in CME-induced MINOCA (Fig. 6). Consistent with the pathogenesis of CME-induced MINOCA, increased LTA4H levels had also been observed in patients with recent or ongoing symptoms of plaque instability [46] and have been linked to thrombosis development [14]. LTB4 in turn has been characterized as a pro-inflammatory mediator promoting leukocyte infiltration and inflammatory responses in infarcted myocardium [22, 27]. Notably, previous studies have identified LTB4-mediated leukotriene signaling as a crucial driver of TNF-α production [15]. TNF-α in turn has been identified as the major cytokine impairing cardiac function following CME [11]. When we stimulated PBMCs isolated 5 h after MINOCA induction with LTB4, we observed an augmented TNF-α release (Fig. 6g). Increased LTB4 levels in CME derived MINOCA may thus accelerate the systemic inflammatory response which may explain our observation of similar systemic cytokine profiles in MINOCA animals when compared to MI animals despite significantly smaller infarction areas.

A recent study investigating a gene expression of CAD and leukotriene signaling with inflammatory cytokines has also delineated a strong interconnectivity with IL-10 [42], which parallels our observation of increased IL-10 levels in MINOCA animals (Fig. 4c). IL-10 has been suggested to regulate LTB4 expression with inhibiting arachidonate 5 lipoxygenase activation proteins [21]. Indeed, inhibiting leukotriene signaling during MI has shown to impede excessive inflammation with reduced IL-1β expression, diminished leukocyte infiltration and to improve cardiac function in a murine model of MI [27]. Similarly, an early study on rabbits demonstrated that pretreatment with a leukotriene synthesis inhibitor ameliorated ECG derangements and reduced mortality following coronary ligation [48]. Considering our observation of increased LTB4 signaling in animals undergoing MINOCA, the potential of leukotriene receptor antagonists which have already been suggested as therapeutic regimens for MI may be of even more relevance for the treatment of MINOCA [2, 41]. Moreover, targeting pro-inflammatory cytokines may be of further therapeutic value as previous clinical trials inhibiting systemic IL-6 expression following MI have achieved significant improvements of cardiac function [4].

However, even with a unique model and findings, our study has several limitations. First, our study lacks transferability to dissection, vasospasm, or microvascular dysfunction types of MINOCA. Second, the experimental animal cohort does not fully reflect the clinical patient population with only young, female and healthy animals included. Notably, male pigs are only attainable as castrated adult animals with compromised translational relevance for human patients. Since, MINOCA is known to occur in younger patients than classic MI [55], we focused on utilizing young pigs to minimize age-specific effects that may confound the data. Lastly, we utilized a healthy animal cohort, which allows an isolated investigation of the effects of CME-derived MINOCA on myocardial and systemic inflammation. However, in the clinical setting, patients may present with partly sever comorbidities that may affect the translational potential of our findings. Therefore, the use of specific porcine strains with more relevant comorbidities such as Ossabaw pigs with propensity to develop metabolic syndrome might reflect the clinical setting more appropriately [13]. Thus, evaluating age and sex, known for being important variables in MINOCA [18, 35, 55], as well as other comorbidities is crucial for future studies aiming to delineate MINOCA diagnostics and therapies.

Lastly, we also acknowledge that the model’s timeline limits our understanding of the leukotriene pathway and general inflammatory differences between MI and MINOCA. Future studies will need to investigate the observed changes at earlier timepoints and in chronic models of CME-induced MINOCA to determine the translational diagnostic and therapeutic relevance. In addition, as shown in our previous publication [8], we acknowledge that our biological model does not recapitulate reduced coronary reserve, as often seen in MINOCA patients and other large animal models [23, 50]. While porcine coronary anatomy renders microvasculature resistance measurement challenging, we also inject a low amount of microthrombi in our model. As a result, the healthy microvasculature can compensate for occluded portions, hence maintaining overall function of the coronary microvasculature during the short-term follow-up in this trial.

In summary, this study dissects inflammatory pathways associated with MINOCA and unveils novel biomarkers for cardiac microembolization within the inflammatory cascade. While we confirm troponin differences, we thus expand on these findings by measuring lower creatine kinase, a smaller infarct size, and less interstitial bleeding in MINOCA animals. We also note higher immune cell infiltration within thrombotic areas in MINOCA animals. While MINOCA animals exhibit higher IL-10, MI animals exhibited higher IFN-γ. The miRNA profile of MINOCA animals also exhibits a dysregulated and augmented inflammatory response, compared to MI which had miRNA markers of ischemic injury. Finally, we utilize proteomics to pinpoint the leukotriene pathway that augments the inflammatory response found in MINOCA. The leukotriene pathway may furthermore serve as a specific therapeutic target to dampen accelerated inflammatory signaling following MINOCA.

## Supplementary Information

Below is the link to the electronic supplementary material.

Supplementary file1 (DOCX 73 KB)

## Data Availability

The data that support the findings of this study are available from the corresponding author upon reasonable request.
