# Supplementary material for: Molecular screening in a translational large animal trial identifies a differential inflammatory response for MINOCA
Source: Basic Res Cardiol. 2025 Jun 13;120(4):657–75. doi: 10.1007/s00395-025-01118-9 (PMC12325550; doi:10.1007/s00395-025-01118-9)
Supplement: Supplementary file 1 — Supplementary file1 (DOCX 73 KB) [file 395_2025_1118_MOESM1_ESM.docx]

**Supplementary Information**

**Molecular Screening in a Translational Large Animal Trial Identifies a Differential Inflammatory Response for MINOCA**

Jasper Iske^1,2,3,4^, Joshua M. Mesfin^5^, Petra Wolint^5^, Miriam Weisskopf^6^, Christien Beez^1,2,7^, Henriette Thau^1,2,7^, Christian T. Stoeck^6^, January M. Weiner^3^, Melanie M. Hierweger^6^, Eva van Gelder^5^, Thorald Stolte^5^, Nuri Ünesen^5^, Ross Straughan^5^, Lucas S. J. Eckholt^1,2^, Nina Trimmel^6^, Dieter Beule^3^, Heike Meyborg^1,2,7^, Timo Z. Nazari-Shafti^1,2,7,8^, Volkmar Falk^1,2,4,5,7^, Maximilian Y. Emmert^1,2,7,9^ and Nikola Cesarovic^1,2,5^

^1^ Deutsches Herzzentrum der Charité (DHZC), Department of Cardiothoracic and Vascular Surgery, Berlin, Germany

^2^ Charité – Universitätsmedizin Berlin, corporate member of Freie Universität Berlin and Humboldt-Universität zu Berlin and Berlin Institute of Health, Berlin, Germany

^3^ Berlin Institute of Health at Charité-Universitätsmedizin Berlin, Berlin, Germany

^4^ DZHK (German Centre for Cardiovascular Research), Partner Site Berlin, Germany

^5^ Department of Health Sciences and Technology, ETH Zürich, Zürich, Switzerland

^6^ Center for Preclinical Development, University Hospital of Zürich, University of Zürich, Zürich, Switzerland

^7^ BIH Center for Regenerative Therapies (BCRT), Berlin Institute of Health at Charité-Universitätsmedizin Berlin, 13353 Berlin, Germany.

^8^ Michael E. DeBakey Department of Surgery, Baylor College of Medicine, Houston, Texas

^9^ Institute for Regenerative Medicine (IREM), University of Zürich, Zürich, Switzerland

Keywords: microcirculation, MINOCA, MI, inflammation, thrombosis, leukotriene

****Corresponding senior author:***

Nikola Cesarovic, PD, DVM, Ph.D.,

Department of Health Sciences and Technology

ETH Zürich

Leopold-Ruzicka-Weg 4, HCP H 12.1

Email: [nikola.cesarovic@hest.ethz.ch](mailto:nikola.cesarovic@hest.ethz.ch)

**Supplementary Figure 1. MI and CME-Derived MINOCA Induce Similar Cytokine Profiles.**

**(a-b)** Serum samples at 0, 150, and 300 minutes following infarction induction in both, MI and MINOCA animals, were collected and subsequently anti-inflammatory cytokines **(a)** were measured, and IL-2, a T-cell specific pro-inflammatory cytokine **(b)**, was measured using multi-ELISA. Data are displayed as mean ± SEM. Statistical significance was determined by a repeated measure 2-way ANOVA (mixed model) with a Geisser-Greenhouse correction and Tukey’s post-hoc correction for multiple comparisons. Abbreviations: MI, classic myocardial infarction; MINOCA, myocardial infarction with non-obstructive coronary arteries. N = 4 for MI, N = 6 for MINOCA.

**Supplementary Table 1. Individual Hemodynamic Parameters and Events.**

| **Systolic Arterial Pressure (SAP; mmHg)** | | | | | | | | | | | | | | | | | | | | |
| --- | --- | --- | --- | --- | --- | --- | --- | --- | --- | --- | --- | --- | --- | --- | --- | --- | --- | --- | --- | --- |
| **Groups** | **Animal no.** | **Baseline** | **45 min of occlusion** | **Reperfusion / Microthrombi injection** | | | | | | | | | | | | | | | | **Events** |
|  |  |  |  | **0**  **min** | | **60**  **min** | | | **120 min** | | | **180 min** | | | **240 min** | | | **300 min** | |  |
| **MINOCA** | 6  (1) | 95 | n/a | 100 | | 102 | | | 105 | | | 95 | | | 95 | | | 100 | |  |
|  | 7  (2) | 90 | n/a | 68 | | 108 | | | 95 | | | 95 | | | 90 | | | 95 | |  |
|  | 8  (3) | 110 | n/a | 112 | | 102 | | | 95 | | | 95 | | | 92 | | | 85 | |  |
|  | 9  (4) | 115 | n/a | 80 | | 100 | | | 100 | | | - | | | 90 | | | 85 | | hypotension during injection;  no recording for 180 min |
|  | 11  (5) | 120 | n/a | 112 | | 108 | | | 102 | | | 100 | | | 100 | | | 100 | | arrhythmia 1 h after injection |
|  | 12  (6) | 100 | n/a | 100 | | 105 | | | 100 | | | 100 | | | 90 | | | 90 | |  |
|  | 34  (7) | 109 | n/a | 79 | | 103 | | | 95 | | | 95 | | | 94 | | | 89 | |  |
| **MI** | 9  (1) | 130 | 130 | 125 | | 110 | | | 108 | | | 98 | | | - | | | - | | no recording, as transport to the MRI (240 min) and during MRI scan (300 min) |
|  | 11  (2) | 95 | 80 | 82 | | 75 | | | 65 | | | 65 | | | - | | | - | | no recording, as transport to the MRI (240 min) and MRI scan (300 min) |
|  | 12  (3) | 85 | 85 | 78 | | 78 | | | 75 | | | 70 | | | 55 | | | 78 | |  |
|  | 13  (4) | 110 | 100 | 105 | | 98 | | | 90 | | | 80 | | | - | | | - | | hypotension 3.5 h after occlusion;  no recording, as transport to the MRI (240 min) and MRI scan (300 min) |
|  | 14  (5) | 110 | 88 | 90 | | 90 | | | 88 | | | 85 | | | 88 | | | 95 | |  |
|  | 15  (6) | 98 | 90 | 90 | | 92 | | | 90 | | | 85 | | | 82 | | | 88 | |  |
|  | 20  (7) | 105 | 85 | 88 | | 95 | | | 90 | | | 88 | | | 75 | | | 72 | | coronary spasm |
| **Diastolic Arterial Pressure (DAP; mmHg)** | | | | | | | | | | | | | | | | | | | | |
| **Groups** | **Animal no.** | **Baseline** | **45 min of occlusion** | **Reperfusion / Microthrombi injection** | | | | | | | | | | | | | | | | **Events** |
|  |  |  |  | **0**  **min** | **60**  **min** | | | **120 min** | | | **180 min** | | | **240 min** | | | **300 min** | | |  |
| **MINOCA** | 6  (1) | 65 | n/a | 60 | 65 | | | 68 | | | 55 | | | 55 | | | 70 | | |  |
|  | 7  (2) | 60 | n/a | 45 | 65 | | | 58 | | | 60 | | | 52 | | | 85 | | |  |
|  | 8  (3) | 72 | n/a | 75 | 60 | | | 55 | | | 52 | | | 52 | | | 48 | | |  |
|  | 9  (4) | 80 | n/a | 50 | 60 | | | 65 | | | - | | | 55 | | | 75 | | | hypotension during injection;  no recording for 180 min |
|  | 11  (5) | 85 | n/a | 55 | 68 | | | 65 | | | 58 | | | 55 | | | 60 | | | arrhythmia 1 h after injection |
|  | 12  (6) | 65 | n/a | 65 | 65 | | | 65 | | | 62 | | | 52 | | | 68 | | |  |
|  | 34  (7) | 42 | n/a | 60 | 60 | | | 58 | | | 57 | | | 57 | | | 53 | | |  |
| **MI** | 9  (1) | 70 | 82 | 75 | 58 | | | 52 | | | 48 | | | - | | | - | | | no recording, as transport to the MRI (240 min) and during MRI scan (300 min) |
|  | 11  (2) | 55 | 45 | 48 | 45 | | | 35 | | | 32 | | | - | | | - | | | no recording, as transport to the MRI (240 min) and MRI scan (300 min) |
|  | 12  (3) | 50 | 50 | 52 | 42 | | | 38 | | | 38 | | | 32 | | | 48 | | |  |
|  | 13  (4) | 65 | 60 | 65 | 55 | | | 50 | | | 40 | | | - | | | - | | | hypotension 3.5 h after occlusion  no recording, as transport to the MRI (240 min) and MRI scan (300 min) |
|  | 14  (5) | 78 | 70 | 65 | 65 | | | 60 | | | 50 | | | 50 | | | 60 | | |  |
|  | 15  (6) | 52 | 55 | 55 | 50 | | | 50 | | | 48 | | | 50 | | | 42 | | |  |
|  | 20  (7) | 65 | 62 | 60 | 55 | | | 52 | | | 50 | | | 45 | | | 40 | | | coronary spasm |
| **Mean Arterial Pressure (MAP; mmHg)** | | | | | | | | | | | | | | | | | | | | |
| **Groups** | **Animal no.** | **Baseline** | **45 min of occlusion** | **Reperfusion / Microthrombi injection** | | | | | | | | | | | | | | | | **Events** |
|  |  |  |  | **0**  **min** | | | **60**  **min** | | | **120 min** | | | **180 min** | | | **240 min** | | | **300 min** |  |
| **MINOCA** | 6  (1) | 80 | n/a | 80 | | | 84 | | | 87 | | | 75 | | | 75 | | | 85 |  |
|  | 7  (2) | 75 | n/a | 57 | | | 87 | | | 77 | | | 78 | | | 71 | | | 90 |  |
|  | 8  (3) | 91 | n/a | 94 | | | 81 | | | 75 | | | 74 | | | 72 | | | 67 |  |
|  | 9  (4) | 98 | n/a | 65 | | | 80 | | | 83 | | | - | | | 73 | | | 80 | hypotension during injection;  no recording for 180 min |
|  | 11  (5) | 103 | n/a | 84 | | | 88 | | | 84 | | | 79 | | | 78 | | | 80 | arrhythmia 1 h after injection |
|  | 12  (6) | 83 | n/a | 83 | | | 85 | | | 83 | | | 81 | | | 71 | | | 79 |  |
|  | 34  (7) | 87 | n/a | 77 | | | 80 | | | 74 | | | 75 | | | 74 | | | 69 |  |
| **MI** | 9  (1) | 100 | 106 | 100 | | | 84 | | | 80 | | | 73 | | | - | | | - | no recording, as transport to the MRI (240 min) and during MRI scan (300 min) |
|  | 11  (2) | 75 | 63 | 65 | | | 60 | | | 50 | | | 49 | | | - | | | - | no recording, as transport to the MRI (240 min) and MRI scan (300 min) |
|  | 12  (3) | 68 | 68 | 65 | | | 60 | | | 57 | | | 54 | | | 44 | | | 63 |  |
|  | 13  (4) | 88 | 80 | 85 | | | 77 | | | 70 | | | 60 | | | - | | | - | hypotension 3.5 h after occlusion;  no recording, as transport to the MRI (240 min) and MRI scan (300 min) |
|  | 14  (5) | 94 | 79 | 78 | | | 78 | | | 74 | | | 68 | | | 69 | | | 78 |  |
|  | 15  (6) | 75 | 73 | 73 | | | 71 | | | 70 | | | 67 | | | 66 | | | 65 |  |
|  | 20  (7) | 85 | 74 | 74 | | | 75 | | | 71 | | | 69 | | | 60 | | | 56 | coronary spasm |
| **Heart Rate (HR; bpm)** | | | | | | | | | | | | | | | | | | | | |
| **Groups** | **Animal no.** | **Baseline** | **45 min of occlusion** | **Reperfusion / Microthrombi injection** | | | | | | | | | | | | | | | | **Events** |
|  |  |  |  | **0**  **min** | | | **60**  **min** | | | **120 min** | | | **180 min** | | | **240 min** | | | **300 min** |  |
| **MINOCA** | 6  (1) | 85 | n/a | 78 | | | 75 | | | 75 | | | 75 | | | 75 | | | 80 |  |
|  | 7  (2) | 60 | n/a | 60 | | | 65 | | | 68 | | | 70 | | | 72 | | | 88 |  |
|  | 8  (3) | 62 | n/a | 62 | | | 60 | | | 62 | | | 65 | | | 68 | | | 68 |  |
|  | 9  (4) | 80 | n/a | 70 | | | 72 | | | 78 | | | 80 | | | 85 | | | 48 | hypotension during injection |
|  | 11  (5) | 65 | n/a | 65 | | | 65 | | | 68 | | | 65 | | | 75 | | | 78 | arrhythmia 1 h after injection |
|  | 12  (6) | 68 | n/a | 75 | | | 75 | | | 75 | | | 68 | | | 65 | | | 68 |  |
|  | 34  (7) | 62 | n/a | 65 | | | 102 | | | 89 | | | 95 | | | 92 | | | 96 |  |
| **MI** | 9  (1) | 60 | 58 | 58 | | | 58 | | | 62 | | | 62 | | | - | | | - | no recording, as transport to the MRI (240 min) and during MRI scan (300 min) |
|  | 11  (2) | 65 | 58 | 60 | | | 60 | | | 65 | | | 68 | | | 67 | | | 74 |  |
|  | 12  (3) | 52 | 62 | 62 | | | 62 | | | 72 | | | 72 | | | 88 | | | 92 |  |
|  | 13  (4) | 65 | 65 | 65 | | | 55 | | | 60 | | | 75 | | | - | | | - | hypotension 3.5 h after occlusion  no recording, as transport to the MRI (240 min) and MRI scan (300 min) |
|  | 14  (5) | 70 | 70 | 65 | | | 72 | | | 75 | | | 68 | | | 72 | | | 80 |  |
|  | 15  (6) | 62 | 60 | 60 | | | 60 | | | 65 | | | 70 | | | 68 | | | 68 |  |
|  | 20  (7) | 68 | 98 | 95 | | | 82 | | | 65 | | | 65 | | | 65 | | | 62 | coronary spasm |

**Supplementary Table 2. MI MRI Parameters**

|  | **R-R[ms]** | **LVM[mL]** | **LVM[g]** | **EDV [mL]** | **ESV [mL]** | **SV [mL]** | **EF [%]** | **CO [L/min]** |
| --- | --- | --- | --- | --- | --- | --- | --- | --- |
| **Average** | 813.4 | 119.7 | 125.7 | 143.2 | 59.3 | 83.9 | 58.6 | 6.4 |
| **SEM** | 46.3 | 5.2 | 5.5 | 5.5 | 3.3 | 4.4 | 1.9 | 0.6 |
